# Supplementary material for: Modeling glioblastoma heterogeneity as a dynamic network of cell states
Source: Mol Syst Biol. 2021 Sep 16;17(9):e10105. doi: 10.15252/msb.202010105 (PMC8444284; doi:10.15252/msb.202010105)
Supplement: Supplementary file 5 — Source Data for Figure 3 [file MSB-17-e10105-s001.zip › Figure3A_sourcedata/GSEA_3065/hallmarks_state5.GseaPreranked.1623416302988/HALLMARK_TGF_BETA_SIGNALING.html]

Details for gene set HALLMARK\_TGF\_BETA\_SIGNALING[GSEA]

|  || Dataset | state5 |
| Phenotype | NoPhenotypeAvailable |
| Upregulated in class | na\_neg |
| GeneSet | HALLMARK\_TGF\_BETA\_SIGNALING |
| Enrichment Score (ES) | -0.4500643 |
| Normalized Enrichment Score (NES) | -1.3576996 |
| Nominal p-value | 0.0727003 |
| FDR q-value | 0.12504053 |
| FWER p-Value | 0.855 |
Table: GSEA Results Summary

  

Fig 1: Enrichment plot: HALLMARK\_TGF\_BETA\_SIGNALING      
 Profile of the Running ES Score & Positions of GeneSet Members on the Rank Ordered List

  

| PROBE | GENE SYMBOL | GENE\_TITLE | RANK IN GENE LIST | RANK METRIC SCORE | RUNNING ES | CORE ENRICHMENT || 1 | BMPR2 |  |  | 1013 | 0.209 | -0.0837 | No |
| 2 | SLC20A1 |  |  | 1035 | 0.206 | -0.0568 | No |
| 3 | SERPINE1 |  |  | 1221 | 0.182 | -0.0517 | No |
| 4 | APC |  |  | 1704 | 0.133 | -0.0868 | No |
| 5 | TGFBR1 |  |  | 1724 | 0.132 | -0.0702 | No |
| 6 | SPTBN1 |  |  | 1880 | 0.120 | -0.0705 | No |
| 7 | BMPR1A |  |  | 2003 | 0.111 | -0.0683 | No |
| 8 | XIAP |  |  | 2072 | 0.107 | -0.0608 | No |
| 9 | TRIM33 |  |  | 2953 | 0.055 | -0.1514 | No |
| 10 | ARID4B |  |  | 3078 | 0.048 | -0.1585 | No |
| 11 | SKIL |  |  | 3291 | 0.037 | -0.1769 | No |
| 12 | SMURF1 |  |  | 3334 | 0.035 | -0.1767 | No |
| 13 | CTNNB1 |  |  | 3417 | 0.031 | -0.1815 | No |
| 14 | TGFB1 |  |  | 3503 | 0.027 | -0.1872 | No |
| 15 | SMURF2 |  |  | 3536 | 0.026 | -0.1871 | No |
| 16 | FURIN |  |  | 3719 | 0.019 | -0.2047 | No |
| 17 | THBS1 |  |  | 3755 | 0.017 | -0.2062 | No |
| 18 | MAP3K7 |  |  | 3835 | 0.014 | -0.2130 | No |
| 19 | SMAD3 |  |  | 3915 | 0.011 | -0.2203 | No |
| 20 | BMP2 |  |  | 4770 | -0.025 | -0.3124 | No |
| 21 | PPM1A |  |  | 4828 | -0.027 | -0.3149 | No |
| 22 | CDK9 |  |  | 5042 | -0.034 | -0.3339 | No |
| 23 | PPP1R15A |  |  | 5321 | -0.045 | -0.3586 | No |
| 24 | NCOR2 |  |  | 5345 | -0.047 | -0.3546 | No |
| 25 | SKI |  |  | 5441 | -0.050 | -0.3582 | No |
| 26 | KLF10 |  |  | 5448 | -0.050 | -0.3517 | No |
| 27 | CDKN1C |  |  | 5823 | -0.066 | -0.3842 | No |
| 28 | HIPK2 |  |  | 6320 | -0.091 | -0.4267 | No |
| 29 | TJP1 |  |  | 6500 | -0.101 | -0.4324 | No |
| 30 | FNTA |  |  | 6659 | -0.111 | -0.4343 | Yes |
| 31 | SMAD1 |  |  | 6662 | -0.112 | -0.4187 | Yes |
| 32 | JUNB |  |  | 6665 | -0.112 | -0.4030 | Yes |
| 33 | IFNGR2 |  |  | 6865 | -0.127 | -0.4073 | Yes |
| 34 | TGIF1 |  |  | 6926 | -0.131 | -0.3954 | Yes |
| 35 | ID2 |  |  | 7333 | -0.171 | -0.4166 | Yes |
| 36 | HDAC1 |  |  | 7459 | -0.187 | -0.4040 | Yes |
| 37 | ID1 |  |  | 7821 | -0.238 | -0.4106 | Yes |
| 38 | PMEPA1 |  |  | 7830 | -0.241 | -0.3774 | Yes |
| 39 | UBE2D3 |  |  | 8163 | -0.314 | -0.3701 | Yes |
| 40 | PPP1CA |  |  | 8193 | -0.324 | -0.3274 | Yes |
| 41 | WWTR1 |  |  | 8384 | -0.383 | -0.2944 | Yes |
| 42 | ID3 |  |  | 8676 | -0.544 | -0.2498 | Yes |
| 43 | FKBP1A |  |  | 8719 | -0.581 | -0.1721 | Yes |
| 44 | RAB31 |  |  | 8831 | -0.687 | -0.0870 | Yes |
| 45 | RHOA |  |  | 8869 | -0.734 | 0.0129 | Yes |
Table: GSEA details [plain text format]

  

Fig 2: HALLMARK\_TGF\_BETA\_SIGNALING: Random ES distribution      
 Gene set null distribution of ES for **HALLMARK\_TGF\_BETA\_SIGNALING**
